# Ellagic acid promoted efficacy of oxaliplatin on wild-type *TP53* colorectal cancer via regulating ataxia-telangiectasia mutated protein kinase and Wip1

**DOI:** 10.3389/fphar.2026.1944826

**Published:** 2026-10-15

**Authors:** Ying Zhao, Mingqian Ma, Siqi Han, Jingxian Hu, Xi Guo, Wei Wang, Shudan Liu, Zhenying Zhao, Yaya Qiao, Yijia Wang

**Affiliations:** 1 Graduate School of Tianjin University of Traditional Chinese Medicine, Tianjin, China; 2 Department of Pathology, Tianjin Union Medical Center, The First Affiliated Hospital of Nankai University, Tianjin, China; 3 Department of Pharmacy, Tianjin Union Medical Center, The First Affiliated Hospital of Nankai University, Tianjin, China; 4 Key Laboratory of Molecular Microbiology and Technology, Ministry of Education, TEDA Institute of Biological Sciences and Biotechnology, Nankai University, Tianjin, China; 5 Ningxia Medical University General Hospital, Yinchuan, Ningxia, China

**Keywords:** ATM, colorectal cancer, ellagic acid, oxaliplatin, Wip1

## Abstract

**Introduction:**

Drug resistance in colorectal cancer frequently leads to chemotherapy failure. The application of small-molecule compounds to enhance chemotherapy efficacy has become a common therapeutic strategy. Ellagic acid, a natural polyphenol in plants, is abundantly contained in herbal medicines used for adjuvant chemotherapy against colorectal cancer. Nevertheless, the regulatory effect of ellagic acid on oxaliplatin-based chemotherapy remains unclear.

**Methods:**

Cell Counting Kit-8 and colony formation assays were used to evaluate the efffcacy of the combination of ellagic acid and oxaliplatin. Network pharmacology, Human Phospho-Kinase Array, co-immunoprecipitation and thermal shift assay were used to screen potential targets of ellagic acid in chemotherapy sensitization. Transfection of overexpression plasmids and shRNAs against these targets was performed to verify the underlying mechanism. Tumor tissues derived from colorectal cancer patients were used to establish PDX models for *in vivo* experiments. All cells and tissues used in this study have wild-type *TP53*.

**Results:**

Cellular experiments demonstrated that ellagic acid exerts a synergistic effect with oxaliplatin. Target screening revealed that ellagic acid inhibits Wip1, thereby upregulating p53 and resulting in the synergistic anti-tumor effect. Further studies demonstrated that ellagic acid interacts to ATM and suppresses ATM phosphorylation, thereby downregulating Wip1 via the ATM/ AMPK/HIPK2 signaling pathway. Data from FOLFOX chemotherapy indicated that high Wip1 expression correlates with poor prognosis. PDX models demonstrated that ellagic acid enhances the therapeutic efficacy of oxaliplatin, particularly in tumors with high Wip1 expression.

**Discussion:**

Our findings indicate that ellagic acid enhances the antitumor efficacy of oxaliplatin in colorectal cancer with wild-type *TP53* and high Wip1 expression.

## 1 Introduction

As one of the most prevalent malignancies worldwide, Colorectal cancer (Wang et al.) creates considerable global health burdens, while China faces a worsening CRC epidemic featured by increasing incidence and prevalence, calling for greater attention to its prevention and control (Wang et al., 2026). CRC is suitable for surgical resection, but patients with advanced CRC still need adjuvant chemotherapy to prevent postoperative recurrence. FOLFOX and CAPOX are mainstream chemotherapy regimens that extend the overall survival of CRC patients to nearly 20 months (Messersmith, 2019). Among these combination regimens, oxaliplatin (Bardakci et al., 2023) interferes with DNA replication and transcription via its platinum atoms. However, CRC develops resistance to OXA through multiple mechanisms, including enhanced DNA repair capacity, upregulated autophagy, increased drug efflux, and so on. For this reason, complementary therapies have been employed to overcome such resistance. A recent report indicated that a novel platinum (Amuzu et al., 2023) prodrug intervenes in resistance in CRC by inducing apoptosis and ferroptosis (Liu Z. et al., 2024). Some ingredients from traditional Chinese medicine, such as Huaier and liensinine, have been reported to reverse OXA resistance in CRC by inhibiting autophagy (Feng et al., 2024a; Feng et al., 2024b). Active components in plants, especially polyphenol compounds, have been widely used in cancer therapy (Yang et al., 2023; Han et al., 2023). Ellagic acid (Augustynowicz et al.) is a natural polyphenol that exists widely in many plants, such as pomegranate, raspberry, strawberry, Chinese tallow, etc. Among these herbs and fruits, tea is an important dietary source of EA in daily life. Ellagitannin is the major existing form of EA in plants (Tolmie et al., 2023), which has a condensed structure and is transformed into EA by gut microbiota. Compared with ellagitannin, EA has lower molecular weight so that it can be easily absorbed.

Furthermore, some traditional Chinese medicines containing EA are commonly used to enhance the chemotherapeutic effect against CRC. For example, Xihuang Pill, a classic TCM formula, is composed of four herbal ingredients: bovis calculus, moschus moschiferus, boswellia carterii and commiphora myrrha. The last one contains EA according to the TCMSP database (old.tcmsp-e.com/tcmsp.php) (Wu et al., 2022). Xihuang Pill is commonly used in combination with FOLFOX to treat CRC and prevent postoperative recurrence (Yu and An, 2017). Cinquefoils, which contain EA, are widely used to manage CRC in Europe and Asia (Augustynowicz et al., 2022).

EA has been confirmed to have a therapeutic effect on several diseases, such as fatty liver (Elseweidy et al., 2022) and diabetes (Maleki et al., 2023). In addition, EA exhibits several antitumor mechanisms. EA downregulates the phosphorylation levels of Erk and Akt in non-small cell lung cancer, breast cancer, and prostate cancer cell lines (Ma et al., 2024; Eskandari et al., 2016). Some reports showed that EA induces apoptosis in cancer by promoting the p53 signaling pathway, but why EA activates p53 has not been elucidated in experiments (Cheshomi et al., 2021; Ramadan et al., 2023). In addition, some studies indicated that EA has no regulatory effect on p53 (Kumar et al., 2016), and even downregulates p53 (Ardah et al., 2021). Therefore, the mechanism by which EA regulates p53 requires further investigation.

OXA and 5-fluorouracil are both core drugs in FOLFOX. Some studies indicated that EA promoted the therapeutic effect of 5-fluorouracil on CRC. EA synergistically potentiated the chemosensitivity to 5-fluorouracil in several CRC cell lines by upregulating apoptotic indexes (Kao et al., 2012). Urolithin A, the primary metabolite of EA produced by gut microbiota, enhanced the effect of 5-fluorouracil in arresting the cell cycle at G2/M phase (González-Sarrías et al., 2015). Some herbs containing EA were also confirmed to sensitize CRC to 5-fluorouracil. A report indicated that co-treatment of *Sanguisorba officinalis* L. radix and 5-fluorouracil significantly elevated the Bax/Bcl-2 ratio, upregulated ROS levels, and induced mitochondrial dysfunction. Several potential active ingredients, including EA, were identified as contributors to these synergistic effects (Liu et al., 2016). However, to our best knowledge, whether EA promotes the effect of OXA remains unknown.

In this study, the synergistic effect of EA and OXA in wild-type *TP53* CRC therapy was investigated using cellular experiments and patient-derived xenograft (Feng et al.) models. The results showed that EA upregulated p53 by targeting the ATM/AMPK/Wip1 signaling pathway, thereby sensitizing CRC cells to OXA. These findings suggest that EA or certain traditional Chinese medicine formulations containing EA may serve as promising adjuvant therapies to improve the efficacy of OXA-based chemotherapy in CRC.

## 2 Materials and methods

### 2.1 Reagents and antibodies

PPMID mAb (abs112970), Cell Counting Kit-8 (Zuo et al., 2023) were purchased from Absin (Wang et al., 2020). p-p38-MAPK(Thr180/Tyr182) pAb (28796-1-AP), β-Actin mAb (66,009-1) were purchased from Proteintech (Wuhan, Hubei, China). p38-MAPK/MAPK14 mAb (BM4439), p53 pAb (PB9008), p-p53 mAb (BM4095), BAX pAb (A00183), BCL2 pAb (A00040-1), p21 pAb (A00145-1), HIPK2 mAb (M01371), ATM mAb (BA0655-2), p-ATM mAb (BM4008), Goat Anti-Rabbit IgG/HRP antibody (SA00001-2), Rabbit Anti-Ki-67 Polyclonal Antibody (PB9026), TUNEL Apoptosis Detection Kit were purchased from Boster Biological Technology (Wuhan, Hubei, China). ATR mAb (A22124), p-ATR (AP1582) mAb were purchased from ABclonal (Wuhan, Hubei, China). AMPKα1/AMPKα2(Thr172) pAb (AF5908), p-AMPKα1 (Thr183)/AMPKα2 (Thr172) pAb (AF5908), Nuclear Protein Extraction Kit and SABC-HRP Kit with Anti-Rabbit IgG (IHC&ICC) were purchased from Beyotime Biotechnology (Shanghai, China). M6620 (HY-13902) was purchased from MedChemExpress (Monmouth Junction, NJ, United States). Annexin V-FITC/PI apoptosis detection kit (Cat. No. E-CK-A211) was purchased from Elabscience Biotechnology Co.,Ltd. (Wuhan, China) and applied for cell apoptosis detection by flow cytometry.OXA was purchased from Jiangsu Hengrui Medicine (Jiangsu, China). EA (Cat.No. T0465, Lot No. 148400) was purchased from TargetMol (Boston, MA, United States). Minimum Essential Medium and Dulbecco’s modified Eagle’s medium were purchased from Gibco (Thermo Fisher Scientific, Waltham, MA, United States).

### 2.2 Cell culture

Human colon cancer cell lines Caco2 and SW48 were provided by the Shanghai Institutes for Biological Sciences, Chinese Academy of Sciences (Shanghai, China). Cells were cultured in minimum essential medium (MEM) with 20% fetal bovine serum (FBS) for Caco2; Dulbecco’s modified Eagle’s medium (DMEM) with 10% FBS for SW48. Both media were supplemented with 1% penicillin-streptomycin. Cells were maintained at 37 °C in a humidified atmosphere containing 5% CO^2^.

### 2.3 Cell viability and apoptosis assays

Cells were seeded in 96-well plates at a density of 6,000 cells per well. After 12 h, they were treated with the indicated drug(s) for another 48 h. Subsequently, 10 μL CCK-8 reagent was added to each well and incubated for 1–4 h before recording the optical density at 450 nm using a microplate reader. The cell viabilities were normalized to those of the control group. The results were analyzed using Calcusyn software to calculate the IC50 values of different drugs and the combination index (CI).

For apoptosis assays, cells (Caco2, SW48) were plated in 6-well plates (1 × 105 cells/well) and cultivated for 12 h. OXA (2 μM), EA (40 μM), and OXA-EA (40 μM) peptides were added. After incubation for 48 h, the cells in each well were rinsed with PBS, and Annexin V-FITC and PI reagent were added (Elabscience, E-CK-A211) according to the manufacturer’s instructions. Cells were assayed on a microplate flow cytometer.

### 2.4 Cell colony formation assay

Cells were seeded into 6-well plates at a density of 700 cells/well. After 14 days of incubation, the resulting colonies were fixed with methanol and stained with crystal violet.

### 2.5 Human Phospho-Kinase array

Protein phosphorylation profiling was performed using the Proteome Profiler™ Array Kit (ARY003B, R&D Systems) according to the manufacturer’s protocol.

### 2.6 Network pharmacology

Target prediction was performed using the SwissTargetPrediction database. Disease targets were retrieved from the GeneCards, OMIM, and DisGeNET databases with “colorectal cancer” as the keyword. For the GeneCards database, only targets with a relevance score greater than the median were selected. The obtained disease targets were merged and deduplicated. The intersection of drug targets and disease-related genes was generated using Venny 2.1.0 software, yielding the overlapping targets. The overlapping target genes were imported into the STRING database with the following parameters: species set as *Homo sapiens*, minimum interaction score >0.4, and all isolated protein nodes hidden, thereby constructing a protein-protein interaction network for drug therapy of the disease. The MCODE plugin in Cytoscape 3.10.0 was subsequently applied to screen core targets according to the Degree value. The node size and color intensity in the network reflected the magnitude of the Degree value. The component-target-disease data were imported into Cytoscape 3.10.0, and the NetworkAnalyzer tool in Cytoscape was used to calculate the topological properties of the network, generating an adjusted “component-target-disease” network regulation diagram. Gene Ontology (GO) functional enrichment analysis of the drug- and disease-related targets was conducted separately using the DAVID database. The P-value was used as the screening criterion, and the top 20 biological terms with the lowest P-values were selected to construct a bar chart.

### 2.7 Plasmid transfection

Cells were transfected with Wip1-shRNA (HSH114457), Wip1 overexpression plasmid (PPL03566-2a), ATM-shRNA (HSH011736), or ATM overexpression plasmid (EX-T7729-M98) (GeneCopoeia, Inc. Rockville, MD, United States) using Lipofectamine 3,000 reagent and Opti-MEM reduced-serum medium (Invitrogen, Waltham, MA, United States) according to the manufacturer’s protocols. Transfection efficiency was determined after 3 days of transfection by observing the fluorescent signals and further confirmed at the protein level by Western blot analysis.

### 2.8 Western blotting

The extracted proteins were quantified using a BCA assay and then separated via sodium dodecyl sulfate-polyacrylamide gel electrophoresis (SDS-PAGE). Subsequently, the separated proteins were electrotransferred onto polyvinylidene fluoride (PVDF) membranes. The membranes were blocked with 5% non-fat milk in Tris-buffered saline with Tween-20 (TBST) for 1 h at room temperature, followed by overnight incubation with specific primary antibodies at 4 °C. After washing with TBST, the membranes were incubated with the corresponding horseradish peroxidase (HRP)-conjugated secondary antibodies for 1 h. Finally, the protein bands were visualized using enhanced chemiluminescence reagents and captured with a digital imaging system.

### 2.9 Histology and Immunohistochemistry (IHC)

Tumor tissue samples were fixed in 4% paraformaldehyde (PFA), embedded in paraffin, and sectioned into 4-μm-thick slices. The sections were deparaffinized using xylene and rehydrated through a graded series of ethanol, followed by antigen retrieval, permeabilization, and blocking. Subsequently, the sections were incubated with primary antibodies overnight at 4 °C, followed by incubation with corresponding secondary antibodies using the streptavidin-biotin complex (SABC) method. The signal was visualized via a 3,3′-diaminobenzidine (DAB) reaction. After mounting, the slices were subjected to microscopic analysis. Terminal deoxynucleotidyl transferase dUTP nick-end labeling (TUNEL) assays were performed using a TUNEL Apoptosis Detection Kit (Boster Biological Technology, Wuhan, China) according to the manufacturer’s instructions.

### 2.10 Hematoxylin and eosin staining

The paraffin-embedded sections were initially baked at 60 °C for 2 h, deparaffinized with xylene, and rehydrated through a graded series of ethanol. Following staining with hematoxylin, the sections were differentiated in acid alcohol and counterstained with eosin. Finally, the slides were dehydrated in graded ethanol, mounted with neutral balsam, and evaluated under a light microscope.

### 2.11 Clinical data of patients with CRC

A total of 71 patients diagnosed with III stage of wild-type *TP53* CRC, who underwent surgical resection and subsequently received the FOLFOX chemotherapy regimen at Tianjin Union Medical Center between 2015 and 2023, were retrospectively included in this study. Based on the Wip1 expression scores obtained from IHC, patients were categorized into high- and low-expression groups. Clinical outcomes, including overall survival (OS) and disease-free survival (DFS), were monitored during the follow-up period to evaluate the prognostic value of Wip1.

### 2.12 Animal studies

Female BALB/c nude mice (4–6 weeks old, specific pathogen-free) were purchased from Beijing Spebiotech Biotechnology (Beijing, China). The PDX model cohort included three patients (utilizing P4 generation models). When the tumor volume reached approximately 100 mm^3^, each PDX case was randomly assigned to the following six groups (n = 5 per group): vehicle control, low-dose EA monotherapy, high-dose EA monotherapy, OXA monotherapy, low-EA + OXA combination therapy, and high-EA + OXA combination therapy.

During the treatment, EA and/or OXA were administered to the PDX models via intraperitoneal (i.p.) injection. The dosage for EA was 100 mg/kg or 200 mg/kg once daily, while OXA was administered at 4 mg/kg every 3 days. Control mice (n = 5) receiving daily vehicle injections served as the baseline group. The treatment lasted for 14 days. On the second day after the end of administration, the mice were euthanized, and tumor tissues were harvested for histological and immunohistochemical (IHC) analyses. During this period, tumor volume and body weight were measured every 3 days using digital calipers and an electronic balance, respectively. To evaluate the efficacy of the treatment regimens, tumor volumes were normalized based on the initial volume at the start of drug intervention (Day 0), and the final tumor volume changes were compared across groups at the end of each treatment stage.

Survival status was monitored for up to 60 days post-inoculation, and animals reaching predefined humane endpoints were euthanized and recorded as events for survival analysis. The euthanasia criteria included a tumor volume >2000 mm^3^ or a maximum tumor diameter >20 mm, body weight loss > 20%, and the appearance of ulceration or severe necrosis.

Anesthesia was induced using isoflurane. A dedicated inhalation anesthetic machine was used to mix pure oxygen for anesthesia, vaporizing the isoflurane via the oxygen flow. A concentration of 2%–3% inhaled isoflurane was used for induction, and 1.5%–2% for maintenance. The anesthesia duration was 2–3 min. When the mouse fell over on its own and its respiration slowed down from a rapid rate, it was removed from the anesthesia chamber, and its corneal and pain reflexes were checked. The cornea was gently touched with the tip of a hemostat; if the mouse did not blink or flinch, it indicated the disappearance of the corneal reflex. Then, the mouse’s toe was clamped with the hemostat using appropriate force; if the mouse showed no struggling or flinching, it indicated the disappearance of the pain reflex. At this point, the mouse was considered fully anesthetized. Whether supplemental anesthesia was needed had to be determined based on the duration of the surgery. The anesthetized mice were euthanized using cervical dislocation. Death was verified by the absence of respiration and heartbeat, as well as fixed and dilated pupils.

### 2.13 Statistical analysis

All statistical analyses were performed using GraphPad Prism 10.0 (San Diego, CA, United States). The Shapiro-Wilk test was applied to assess the normality of datasets. Data were presented as mean ± standard error of the mean (SEM) unless otherwise specified. Two-group comparisons were carried out using two-tailed Student’s t-test or Mann-Whitney U test. For multiple-group comparisons covering protein expression, immunohistochemical MOD scores, one-way analysis of variance (ANOVA) followed by Tukey’s *post hoc* test was used for normally distributed data with homogeneity of variance; the Kruskal–Wallis test with Dunn’s *post hoc* test was adopted for non-normally distributed datasets.

For single-time-point dose-response experiments such as CCK-8 cell-viability assays, one-way ANOVA was performed followed by either Dunnett’s multiple comparisons test (multiple treatment groups versus a single control) or Tukey’s *post hoc* test for all-pairwise comparisons; Dunnett’s T3 test was applied for pairwise comparisons when variance was heterogeneous. Two-way ANOVA with Tukey’s *post hoc* test was used for pairwise comparisons of longitudinal *in vivo* tumor-growth data. Mixed-effects models were applied to longitudinal tumor-volume measurements to account for within-mouse repeated-measures correlation.

Non-linear four-parameter sigmoidal regression was performed independently to fit ITDR-CETSA dose-response curves and calculate target-engagement EC_50_ values, which cannot be derived from ANOVA outputs. Correlation analyses were conducted using Pearson’s correlation for normally-distributed data or Spearman’s rank-order correlation for non-normal data. Survival curves were generated by the Kaplan-Meier method, and inter-group survival differences were evaluated with the Log-rank (Mantel-Cox) test. Categorical variables were analyzed using the χ^2^ test or Fisher’s exact test as appropriate. A two-tailed p-value < 0.05 was considered statistically significant.

## 3 Results

### 3.1 Combination treatment of EA and OXA exerts a synergistic effect in CRC cells

Two CRC cell lines with wild-type *TP53*, Caco-2 and SW48, were used to determine whether EA promotes cytotoxicity of OXA. As shown in CCK-8 results of Figure 1A, EA significantly inhibited cell proliferation. Further combination index analysis revealed that the combination of EA and OXA exerted a synergistic effect. As a potential adjuvant agent, side effects are an important factor to be considered. Therefore, the normal colon epithelial cell line NCM460 was used to evaluate the cytotoxicity of EA in normal cells. Based on these results, 40 μM EA and 2 μM OXA were chosen for subsequent *in vitro* experiments. At these concentrations, EA inhibited the growth of NCM460 cells by approximately 10%, while OXA exerted significant cytotoxicity in Caco-2 and SW48 cells. Annexin V-PI flow-cytometry results of Figure 1B show that the combination-treatment group exhibits the highest apoptotic rate. The results of three independent flow cytometry assays are shown in Supplementary Figure S1. The colony formation assay (Figure 1C) further confirmed that EA sensitized CRC cells to OXA at the indicated concentrations. PI flow cytometric analysis of the cell cycle (Supplementary Figure S2) demonstrates that OXA induces prominent G2/M arrest, while combination treatment triggers more apoptosis.

**FIGURE 1:**
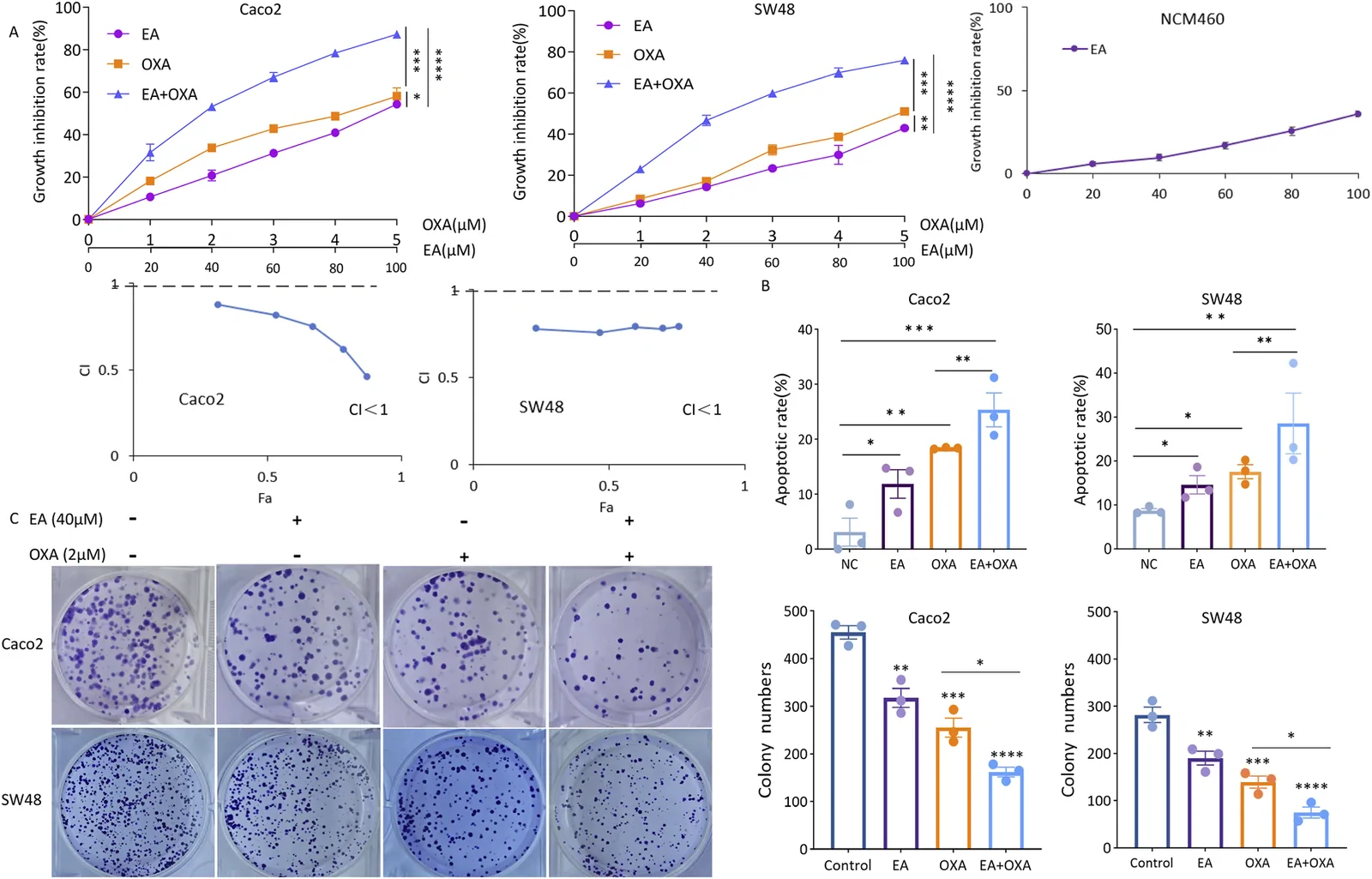
Effect of the combination treatment of EA and OXA on Caco-2, SW48 and NCM460 cells. **(A)** CCK-8 assay results. Cells were treated with EA and OXA alone or in combination at the indicated concentrations. Inhibition rates were compared between monotherapy and combination groups. Combination indices were calculated using CalcuSyn software. **(B)** Annexin V-PI flow cytometry was used to detect cellular apoptosis. **(C)** Colony formation assay results showing proliferative inhibition. Each group was compared with the ‘control’ group, and the ‘OXA’ group was compared with the ‘EA + OXA’ group. Statistical significance: ns, not significant; *p < 0.05; **p < 0.01; ***p < 0.001 and ****P < 0.0001.

### 3.2 EA upregulated p38 and p53 signaling

The chemical structure of EA is shown in Figure 2A. EA (C_14_H_6_O_8_) is a polyphenolic dilactone with a trans-gallic acid tannin structure. Its two lactone rings and four hydroxyl groups endow EA with potent antioxidant activity (Wang et al., 2022). Therefore, a Human Phospho-Kinase Array Kit was used to further screen potential targets of EA associated with oxaliplatin (OXA) resistance. The results presented in Figure 2C demonstrated that EA upregulated p-p53 and p-p38, while inhibiting p-Erk1/2 and p-Chk2. Based on the upregulation of both p-p53 and p-p38, we speculated that Wip1 was inhibited by EA. Wip1, whose full name is wild-type p53-induced phosphatase 1, is encoded by the oncogene PPM1D.

**FIGURE 2:**
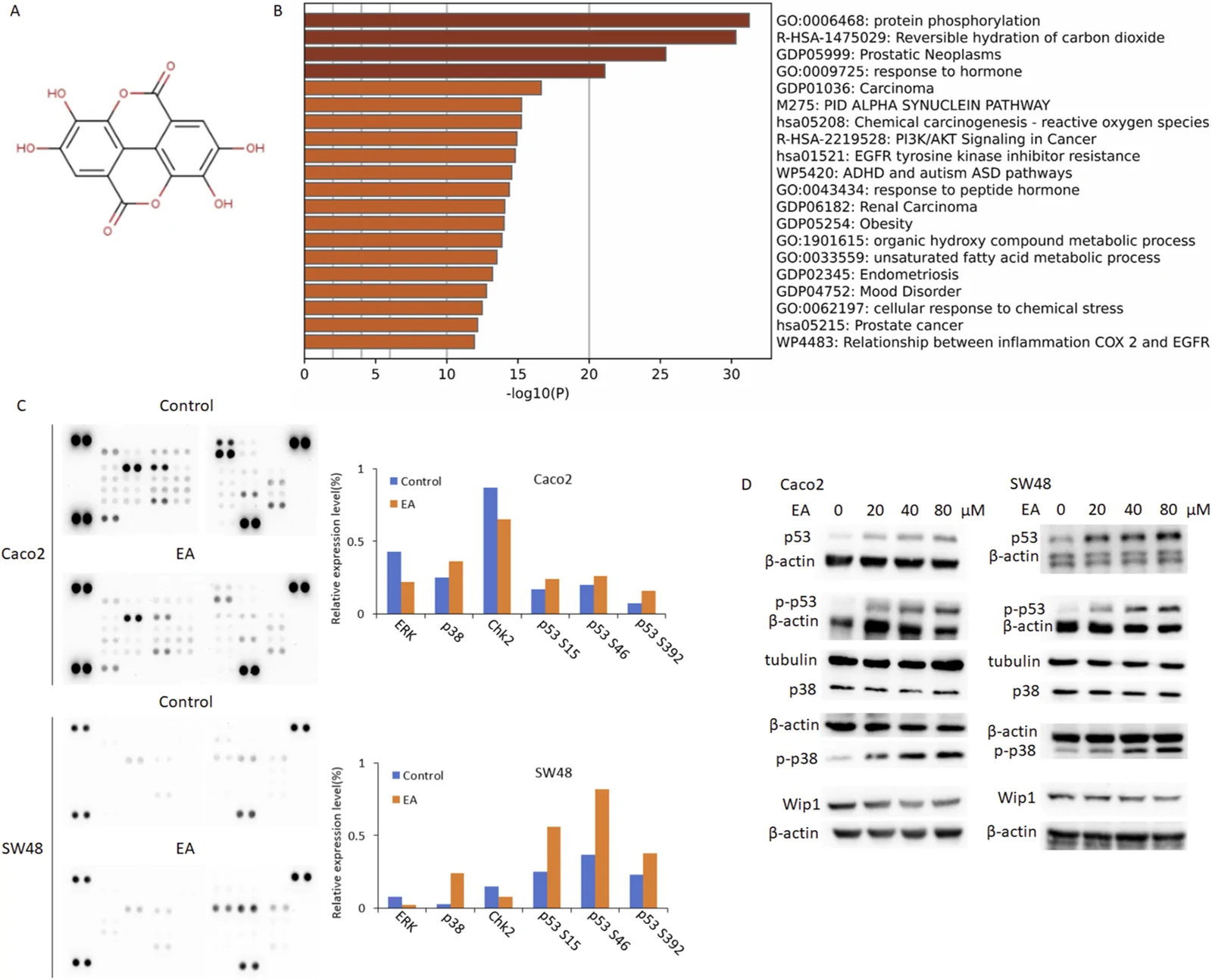
Screening the targets of EA in CRC. **(A)** Chemical structure of EA. **(B)** Network pharmacology analysis. **(C)** Results of the Human Phospho-Kinase Array, including the array images and quantitative grayscale analysis. **(D)** Western blotting analysis of Wip1, p53, and p38 expression in cells treated with gradient concentrations of EA. Drug-treated groups were compared with the control.

As shown in Figure 2B, network pharmacology was applied to predict the potential targets of EA, and the results revealed that protein phosphorylation represented the most prominent regulatory effect of EA. Therefore, a Human Phospho-Kinase Array Kit was used to further screen potential targets of EA associated with OXA resistance. The results presented in Figure 2C demonstrated that EA upregulated p-p53 and p-p38, while inhibiting p-Erk1/2 and p-Chk2. Based on the upregulation of both p-p53 and p-p38, we speculated that Wip1 was inhibited by EA. Wip1, whose full name is wild-type p53-induced phosphatase 1, is encoded by the oncogene *PPM1D* (Wang et al., 2020). There is a negative feedback loop between p53 and Wip1: p53 promotes Wip1 expression, whereas Wip1 in turn suppresses p53 signaling. In addition, Wip1 inactivates p38 by dephosphorylating it at the Thr182 site (Chen et al., 2020). Accumulating evidence suggests that Wip1 negatively regulates p38 activity in various cancer cells by dephosphorylation (Tan et al., 2019). Further Western blotting was performed as shown in Figure 2D, which demonstrated that EA inhibited Wip1 and upregulated p-p53 and p-p38 in a dose-dependent manner. The grayscale value analysis is shown in Supplementary Figure S3. However, molecular docking analysis revealed no direct binding between EA and Wip1 (data not shown).

### 3.3 Synergistic effect of EA and OXA on p53 signaling

As a platinum-based drug, OXA binds to DNA via platinum coordination, resulting in DNA damage. As shown in Figure 3A, the ATM/ATR/p53 signaling pathway (activated by DNA damage) was examined in cells treated with EA and OXA. The grayscale value analysis of Figure 3A is shown in Supplementary Figure S4. The results demonstrated that EA inhibited p-ATM in a dose-dependent manner. EA and OXA synergistically upregulated p53, p21, and the Bax/Bcl-2 ratio. Combined treatment with EA and OXA also significantly inhibited p-ATM and enhanced p-ATR. On the basis of these results, we speculated that OXA upregulates p53 signaling via ATR when ATM is suppressed by EA. ATR and ATM kinases both belong to the phosphatidylinositol 3-kinase-related kinase family. ATM is activated by DNA double-strand breaks (DSBs) to mediate DNA damage repair. The ATM pathway is dysregulated in many types of tumors. As a compensatory survival pathway in response to DNA damage, the ATR-CHK1 axis can be upregulated by such defects (Wang et al., 2024). A negative feedback loop between ATM and ATR was also identified, which is involved in OXA resistance (CombèS et al., 2019). This may explain why EA upregulated p-ATR.

**FIGURE 3:**
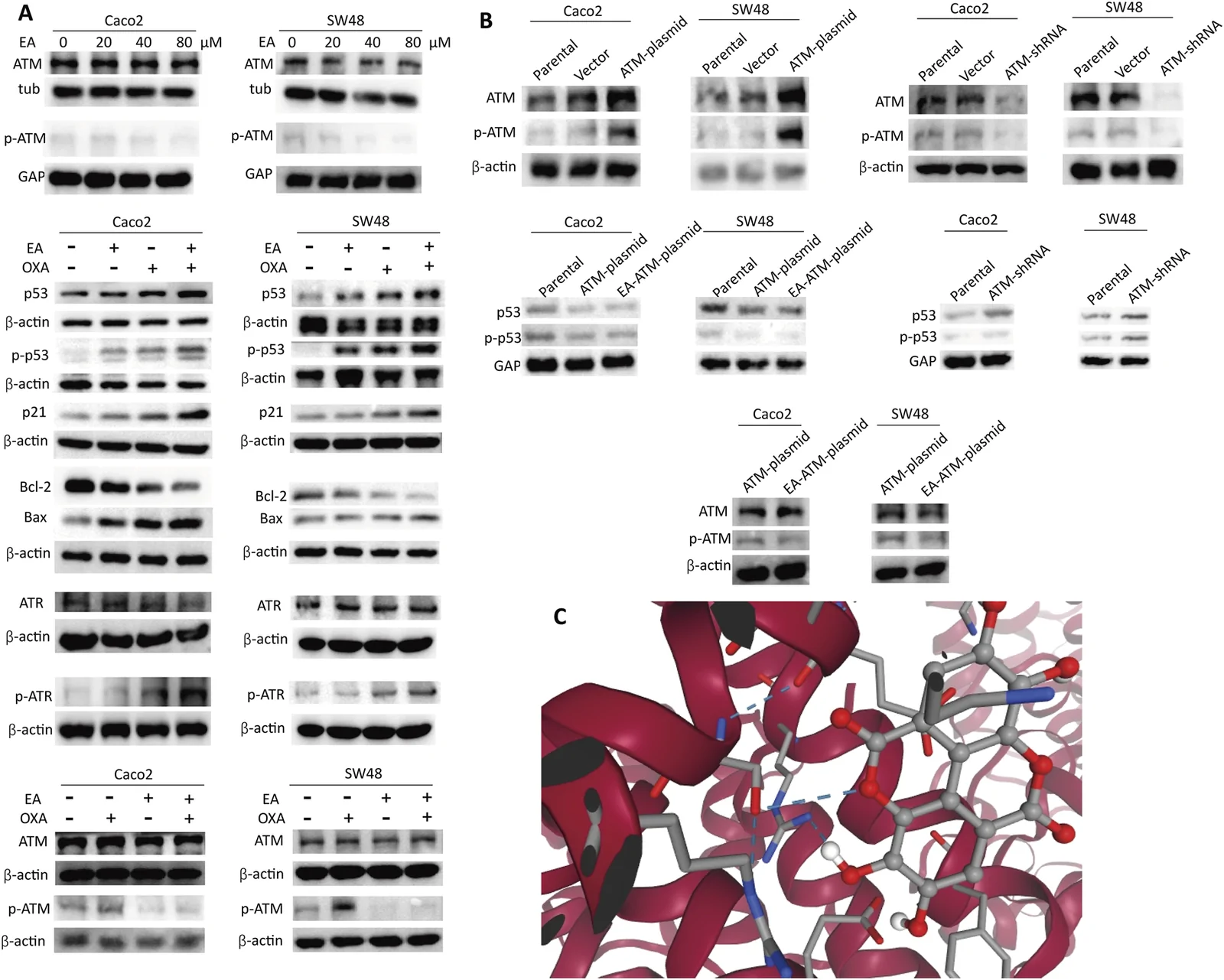
Analysis of ATM/ATR/p53 signaling pathway in EA and OXA treated cells. **(A)** Western blotting results of ATM/ATR/p53 signaling pathway, and its downstream factors Bax/Bcl-2 and p21. **(B)** Western blotting results of ATM/ATR/p53 in ATM plasmid and shRNA transfected cells. **(C)** Molecular docking of EA and ATM.

ATM overexpression plasmid and ATM shRNA transfection were performed to investigate the regulatory mechanism of EA on p53. As shown in Figure 3B, transfection efficiently regulated ATM expression. The grayscale value analysis of Figure 3B is shown in Supplementary Figure S5. Furthermore, transfection of ATM shRNA increased p-ATR and p53 levels, mimicking the effect of EA. Molecular docking analysis (Figure 3C) revealed that EA binds to ATM at residues SER1801 and ARG2279.

In silico simulation and co-immunoprecipitation assays between ATM and biotin-conjugated EA further verified that EA directly targets ATM (Figure 4A). The structure of biotin-conjugated EA is shown in Supplementary Figure S6. Cellular Thermal Shift Assay (CETSA) was applied to further verify the interaction between EA and ATM. As shown in Supplementary Figure S7, EA altered the thermal stability of ATM. CCK-8 assays were performed to confirm that EA enhances the anti-tumor efficacy of OXA by inhibiting ATM. As shown in Figure 4B, ATM overexpression abolished the synergistic effect of EA and OXA. Figure 4C demonstrates that ATM knockdown attenuated OXA resistance, mimicking the effect of EA treatment. The ATR inhibitor M6620 was used to determine the contribution of ATR-dependent compensatory mechanism to the synergistic effect of combination therapy. As shown in Supplementary Figure S8, M6620 (24 h treatment at 200 nmol/L) efficiently reduced p-ATR levels in ‘EA + OXA’ treated cells and abolished the synergistic effect of the combination treatment.

**FIGURE 4:**
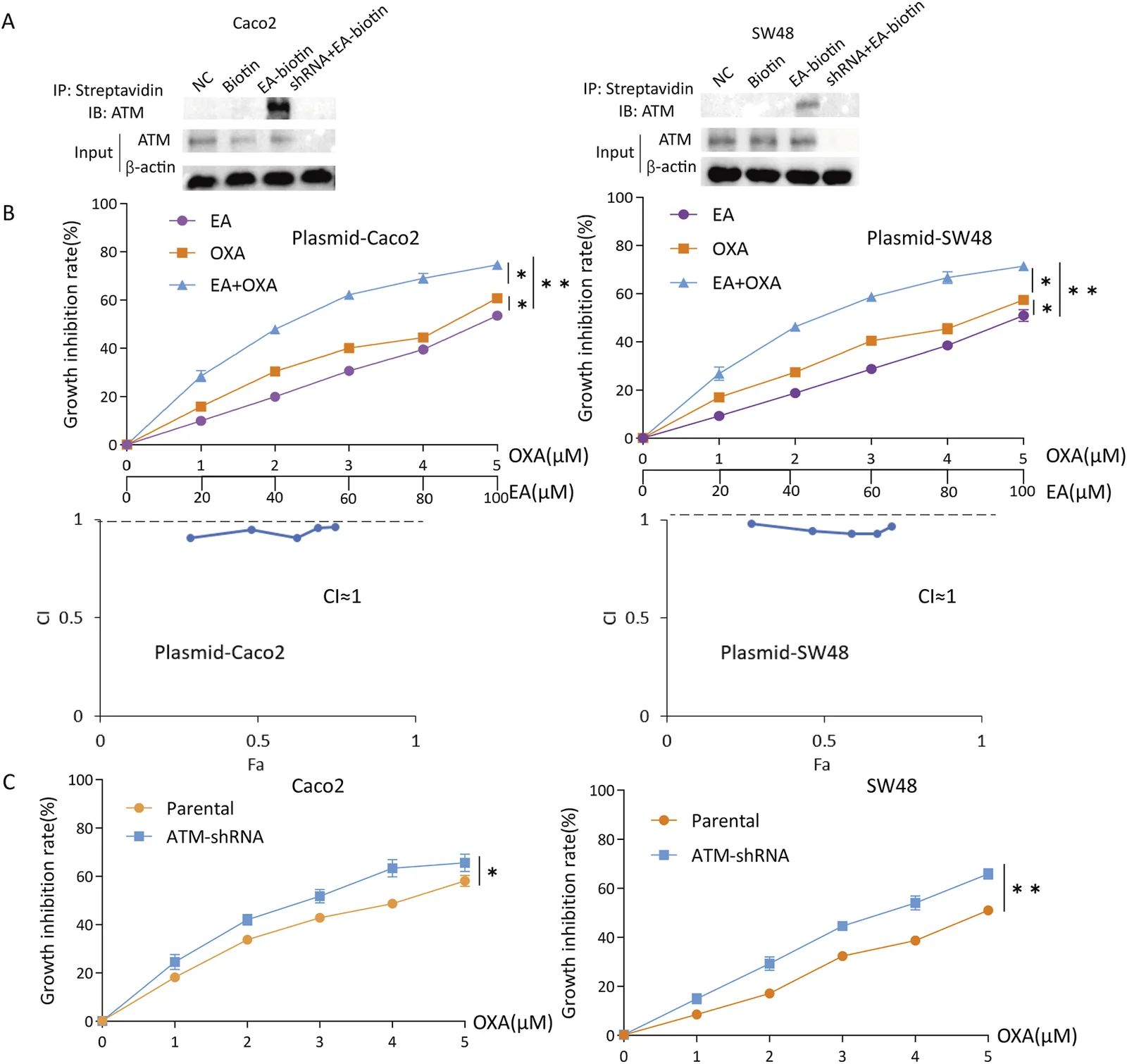
Bind of EA to ATM and its effect on oxaliplatin resistance. **(A)** Immunoprecipitation results showing the interaction between EA-biotin and ATM. **(B)** CCK-8 assay results and CalcuSyn software analysis in cells transfected with ATM overexpression plasmid. **(C)** CCK-8 assay results in ATM shRNA-transfected cells. Data for parental cells are identical to those in the ‘OXA’ group in Figure 1A. Statistical significance: *p < 0.05; **p < 0.01.

### 3.4 EA increased p53 signaling through ATM/AMPK/HIPK2/Wip1 pathway

Although ATM is a key regulator of the cellular response to DNA damage and genome stability, accumulating evidence indicates that ATM also modulates energy metabolism by phosphorylating AMPK (Shimura et al., 2024; Shen et al., 2020). After phosphorylation at Thr112 site by ATM, AMPK facilitates phosphorylation of HIPK2 in Thr112, Ser114 and Thr1107 (Choi et al., 2013). HIPK2 is a member of the nuclear protein kinase family and binds to Wip1. This interaction promotes the phosphorylation of Wip1 at Ser54/85, leading to its ubiquitylation and proteasomal degradation. Phosphorylated HIPK2 translocates to the cytoplasm and loses its ability to bind Wip1 (Nahta and Castellino, 2021; Apaydin et al., 2023). According to these reports, the AMPK/HIPK2/Wip1 pathway in EA-treated cells was analyzed in Figure 5A. The results showed that EA inhibited p-AMPK and increased the nuclear expression of HIPK2 (the results of EA inhibiting Wip1 are shown in Figure 2D). To confirm the role of ATM in the regulatory effect of EA on Wip1, cells transfected with ATM plasmid or shRNA were subjected to Western blot analysis as shown in Figure 5B. The results indicated that transfection with ATM plasmid reversed the regulatory effect of EA on Wip1, while transfection with ATM shRNA suppressed p-AMPK and Wip1, and increased HIPK2 in the nucleus. Wip1 plasmid and shRNA were transfected into cells to determine the role of Wip1 in the EA-mediated regulation of p53 signaling. Figures 5C,D suggested that EA failed to regulate Wip1 and p53 in cells transfected with Wip1 plasmid, whereas p53 was upregulated in cells transfected with Wip1 shRNA. The grayscale value analysis of Figures 5A–D is shown in Supplementary Figure S9. The mechanism of this synergistic effect is shown in Figure 5E. Further CCK-8 assays in Figures 5F,G demonstrated that overexpression of Wip1 abrogated the synergistic effect of EA and OXA, while knockdown of Wip1 enhanced the efficacy of OXA. Co-immunoprecipitation assays of Supplementary Figures S10A,B further confirmed that the interaction between HIPK2 and Wip1 persists under EA treatment. Ubiquitination analysis of Wip1 (Supplementary Figure S10C) revealed that EA enhances its ubiquitination level.

**FIGURE 5:**
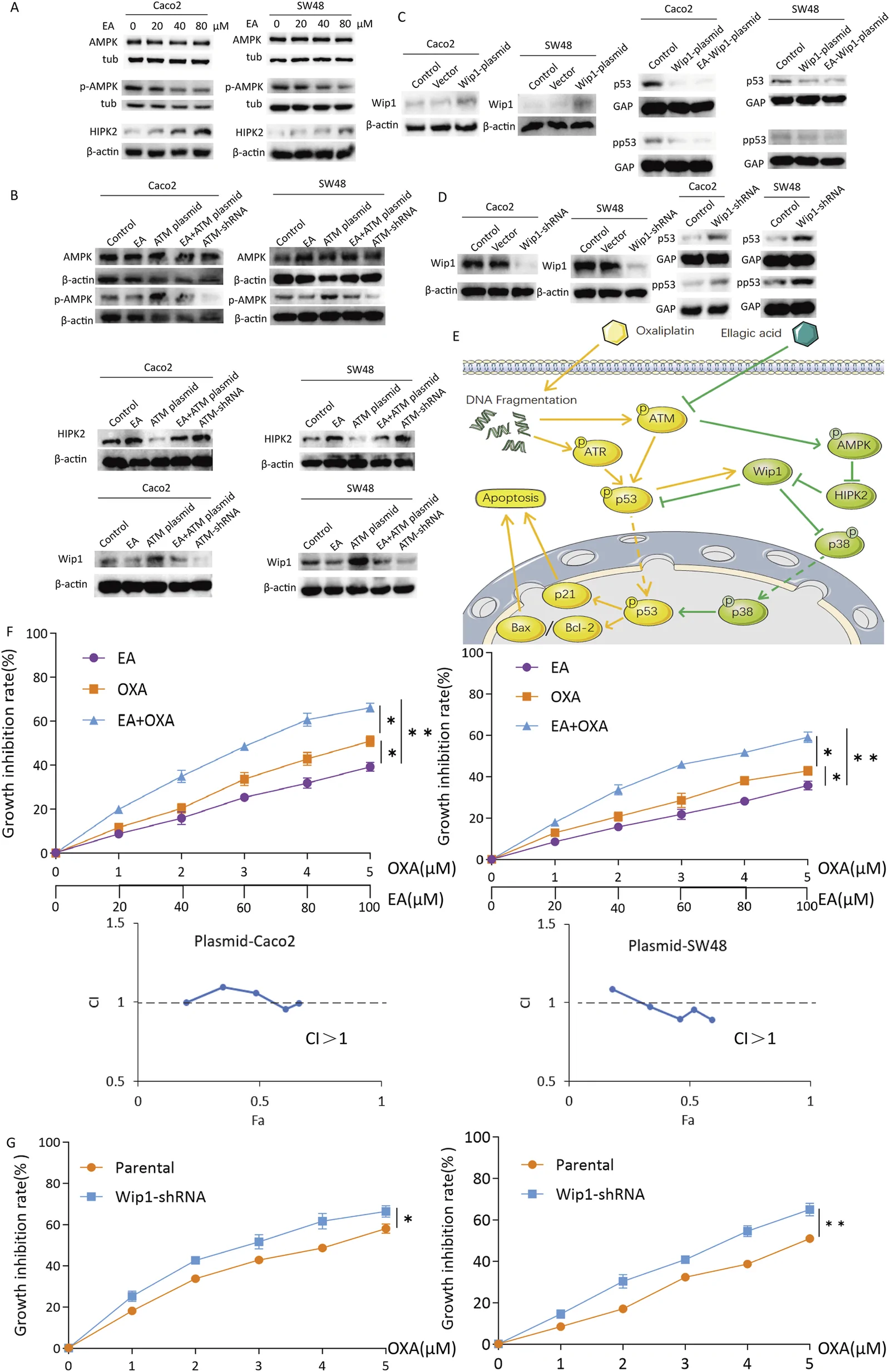
Western blotting analysis of the ATM/AMPK/HIPK2/Wip1 pathway and corresponding functional validation. Cell types are indicated in the top row. **(A)** Western blotting results of p-AMPK, AMPK and HIPK2 in EA-treated parental cells. **(B)** Western blotting results of p-AMPK, AMPK, HIPK2 and Wip1 in ATM shRNA-transfected cells and EA-treated ATM plasmid-transfected cells. **(C)** Western blotting results of Wip1, p-p53 and p53 in Wip1 plasmid-transfected cells. **(D)** Western blotting results of Wip1, p-p53 and p53 in Wip1 shRNA-transfected cells. **(E)** Mechanistic diagram of the synergistic mechanism of EA combined with OXA. **(F)** CCK-8 assay results and CalcuSyn software analysis in Wip1 plasmid-transfected cells. **(G)** CCK-8 assay results of Wip1 shRNA-transfected cells. The data of parental cells are consistent with those of the “OXA” group in Figure 1A. Statistical significance: *p < 0.05; **p < 0.01.

### 3.5 High level of Wip1 relates to low prognosis of FOLFOX treatment

CRC tissues were obtained from 71 patients with stage III disease who received postoperative FOLFOX chemotherapy, and were used to examine Wip1 and ATM expression. Notably, all tissues were collected during surgery and harbored wild-type p53. As shown in Figure 6A, IHC staining results for Wip1 and ATM were each divided into two groups based on median expression values. Wip1 expression was significantly different between the two groups, whereas ATM expression was not. Patient data were stratified into two groups according to Wip1 expression levels. OS and DFS analysis (Figure 6B) indicated that high Wip1 expression correlates with poor prognosis. Patient characteristics are summarized in Supplementary Table S1. In addition, plasma samples from a patient treated with Xihuang Pill were analyzed by LC-MS, and EA was identified as a detectable component in the circulation, as shown in Supplementary Figure S11.

**FIGURE 6:**
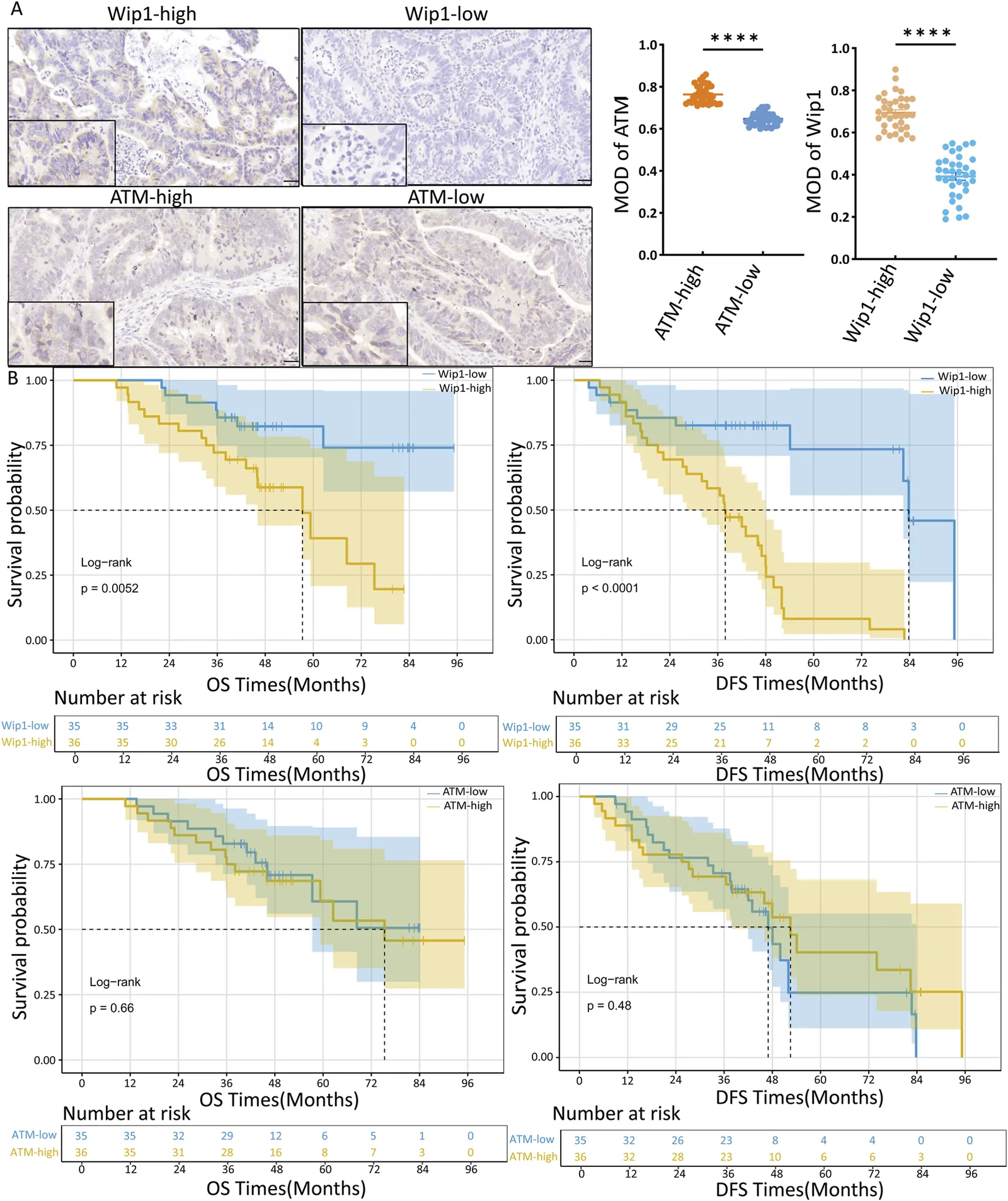
IHC results in CRC tissues and the association between Wip1 expression and prognosis of FOLFOX-treated CRC patients. **(A)** IHC staining for Wip1 and ATM in CRC tissues. **(B)** Kaplan-Meier curves showing overall survival (OS) and disease-free survival (DFS) in patients stratified according to low or high expression of Wip1 and ATM.Statistical significance: ****P < 0.0001.

### 3.6 EA enhances the therapeutic effect of OXA on the PDX model

Two CRC tumors with different Wip1 expression levels were selected to establish PDX models. Whole-exome sequencing results are shown in Supplementary Figure S12 Somatic mutation analysis was performed on representative P2 tumors and the corresponding patient-derived parental tumors. P2 tumors exhibited a high degree of genomic stability, similar to the parental tumors. Figure 7 shows the group-based treatment administration for the PDX model (Figure 7A), body weight change curves (Figure 7B), survival curves (Figure 7C), and tumor volumes at the time of sacrifice (Figure 7D). These results indicate that an appropriate dose of EA enhances the efficacy of OXA *in vivo* in tumor tissues with high Wip1 expression, whereas no significant effect was observed in Wip1-low-expressing tumor tissues. IHC results for Ki67, p53, Wip1, and TUNEL staining analyses are shown in Figure 8. It can be seen that in PDX1 (highly expressing Wip1), EA effectively downregulated Wip1 expression, activated the p53 signaling pathway, and enhanced the antitumor efficacy of OXA, as reflected by Ki-67 and TUNEL staining. However, in PDX2 with low Wip1 expression, EA did not significantly increase p53 activity. Although the combination treatment exhibited the strongest antitumor effect according to Ki-67 and TUNEL results, IHC is not an ideal quantitative method for determining the synergistic effect of drugs. It should be noted that All mice in the ‘200 mg/kg/day EA + 4 mg/kg/3 days OXA’ group died within 2 weeks due to severe drug toxicity, so they are not shown in the figure. Accordingly, drug-related toxicity was re-evaluated. As shown in Supplementary Figure S13, hepatic and renal toxicity was assessed using H&E staining and serological assays. Hepatic injury gradually aggravates in combination groups when the dosage of EA exceeds 160 mg/kg/day, whereas no obvious hepatic or renal toxicity was detected in the other groups. Therefore, we preliminarily consider the regimen of 100 mg/kg/day EA plus 4 mg/kg/3 days OXA to be relatively safe and therapeutically effective. The statistical results of Figure 8 are shown in Supplementary Figure S14.

**FIGURE 7:**
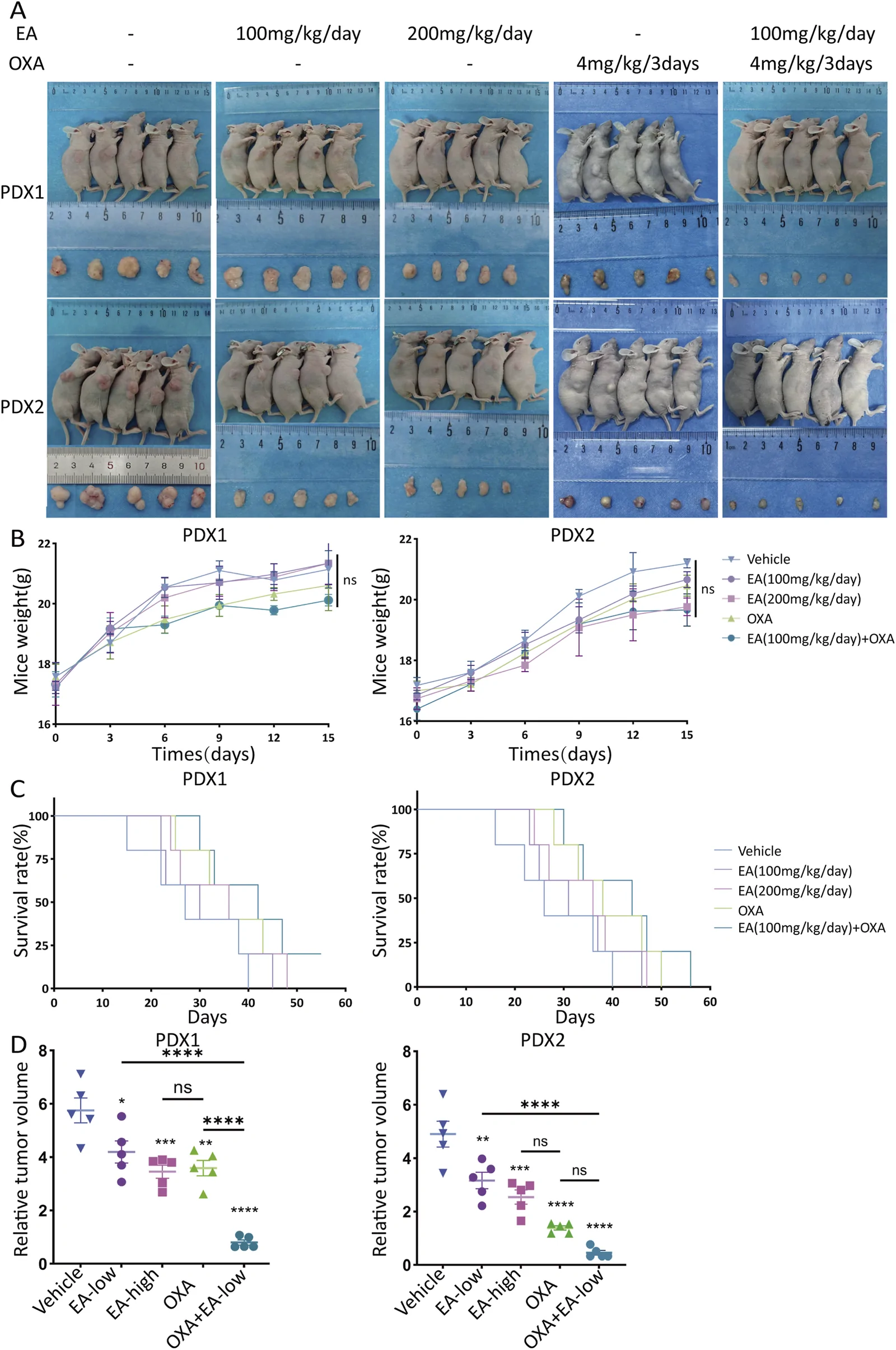
Results of two PDX models. **(A)** Images of mice and tumors from the two PDX models at the time of sacrifice. **(B)** Body weight curves of mice after treatment. **(C)** Survival curves of mice. Kaplan-Meier survival analysis was performed to compare “OXA” versus “EA (100 mg/kg/day)+OXA”. Log-rank test yielded *P* = 0.0076 for PDX1 and *P* = 0.0554 for PDX2. **(D)** Comparison of tumor volumes among different treatment groups. Statistical significance: ns, not significant; *p < 0.05; **p < 0.01; ***p < 0.001 and ****P < 0.0001.

**FIGURE 8:**
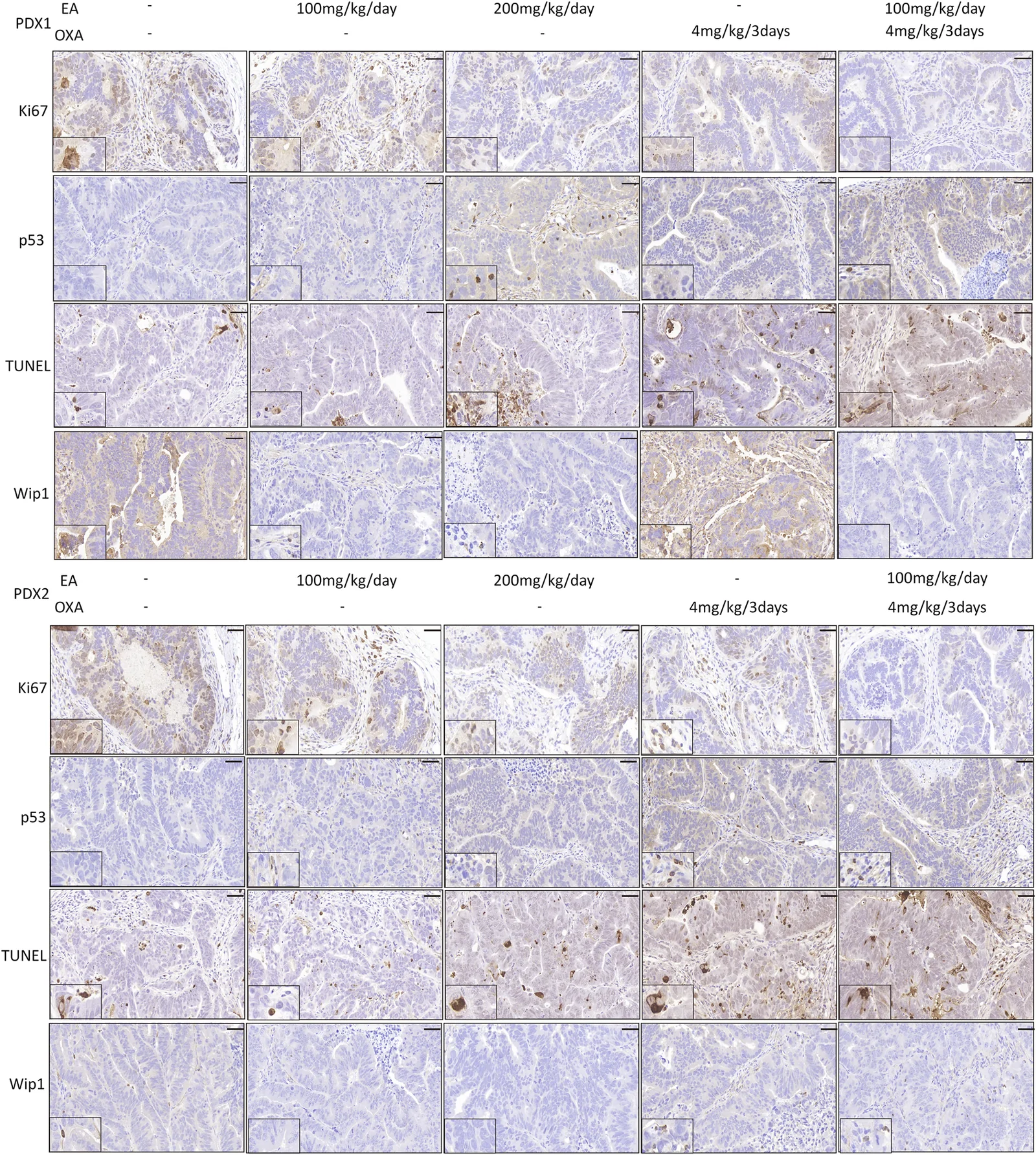
IHC staining for Ki67, p53 and Wip1, as well as TUNEL staining, in two PDX tumor tissues. Different drug combinations are indicated at the top of each panel, and the staining targets are labeled on the left.

## 4 Discussion

Gene mutations in colorectal cancer affect the efficacy of various drugs. Therefore, to minimize confounding factors, this study investigated the mechanism by which EA enhances the therapeutic efficacy of OXA exclusively in the context of wild-type *TP53*. Dysfunctional p53 is common in CRC, with *TP53* mutations occurring in 43% of tumors (Li et al., 2024). *TP53* mutations also influence the choice of combination therapy. For CRC with wild-type *TP53*, combination strategies mainly focus on activating pathways such as oxidative stress, autophagy, and endoplasmic reticulum stress.

*TP53* mutations critically regulate Wip1 expression. Under platinum drug-induced DNA damage, wild-type p53 markedly induces Wip1 expression. Wip1 thereby promotes oxaliplatin resistance via the Wip1–p53 negative feedback loop. In p53-mutant tumors, by contrast, Wip1 is activated only through p53-independent pathways, which alters the relationship between Wip1 and drug resistance (Yang et al., 2018). Currently, several p53-independent modulators of Wip1 are known, including estrogen receptor, c-Jun, and NF-κB (Park et al., 2012). Therefore, only p53 wild-type tumors were investigated in this study to minimize interference from other factors.

The relationship between ATM expression and prognosis remains unclear and may be associated with tumor location and histological type (Lee, 2024). ATM is also a double-edged sword in chemotherapy resistance. Platinum-based drugs induce ATM upregulation, which enhances DNA damage repair and promotes cell survival. Meanwhile, ATM activates the p53 signaling pathway, thereby triggering p53-dependent apoptosis. The impact of ATM inhibition on tumor drug resistance is complex. For example, the ATM inhibitor KU55933 blocks the ATM/Chk2/p53 pathway, thereby reducing the apoptosis of OXA-treated gastric cancer cells (Wu et al., 2020). However, another study reported that inhibiting ATM with KU55933 disrupts the DNA damage repair pathway (Lin et al., 2012). Another study showed that high levels of p-ATM are associated with poor prognosis in glioblastoma, and KU55933 represents a promising therapeutic strategy (Xie et al., 2024). On the other hand, ATR, another upstream factor of p53, exhibits cross-regulation with ATM. A study reported that caffeine inhibits ATR, thereby preventing cells from entering S/G2 arrest to repair DNA damage. This effect results in more DNA double-strand breaks, which in turn activates ATM (Wang et al., 2015). And our study showed that EA inhibits p-ATM while promoting p-ATR, which may also be due to this mechanism.

Several studies have reported compensatory crosstalk between ATM and ATR within the DNA-damage-response (DDR) network, whereby perturbation of one kinase can trigger adaptive changes in the activity of the other. For instance, a synthetic ATM inhibitor elevated p-ATR level significantly (Liu T. T. et al., 2024). Another study on platinum-based chemotherapy showed that the ATR inhibitor M6620 increased p-ATM and p-p53 levels (Turchick et al., 2023). Furthermore, treatment with the ATR inhibitor VE-822 was sufficient to upregulate both p-p53 and p-ATM (D'Aniello et al., 2019). Collectively, these published observations support the notion that pharmacological suppression of one DDR kinase can lead to compensatory activation of the other, and that p53 activation does not strictly require canonical ATM signalling.

Our results indicated that EA upregulates p53. In theory, if p53 upregulation is a trigger, Wip1 expression should increase. However, our data showed that EA inhibits Wip1, which suggests other potential mechanisms. Therefore, we hypothesized that ATM phosphorylates AMPK (Shen et al., 2020; Shimura et al., 2024) to promote Wip1 via the AMPK/HIPK2 pathway (Choi et al., 2013; Nahta and Castellino, 2021; Apaydin et al., 2023). Our results validate this. Wip1 acts as a negative regulator of the DNA damage response by downregulating key factors (including ATM, CHK1/2, p53, and γH2AX) to disrupt DNA damage signaling. It also plays critical roles in immune cell development, hematopoietic stem cell homeostasis, as well as reproductive and neural development. Abnormal Wip1 expression drives malignant phenotypes and mediates therapeutic resistance in several cancer types (Nahta and Castellino, 2021). For example, a study reported that overexpression of Wip1 in osteosarcoma is associated with increased distant metastasis and poor prognosis (He et al., 2021). Another study demonstrated that Wip1 contributes to chemoresistance by suppressing p53 and p38, which is associated with poor prognosis in advanced-stage ovarian clear cell carcinoma (Xu et al., 2022).

Several studies have demonstrated that Wip1 inhibitors exhibit weak anti-tumor effects as single agents but potent efficacy in combination therapy. For example, GSK2830371, a Wip1 inhibitor, shows little anti-tumor effect when used alone. However, it can be combined with an MDM2 inhibitor to co-activate the p53 pathway for the treatment of advanced intrahepatic cholangiocarcinoma (Wu et al., 2021). Another study indicated that the combination of GSK2830371 and a PARP inhibitor can enhance DNA damage, which is used for the treatment of hepatocellular carcinoma (Wu et al., 2021).

Our results indicated that ATM shows little change in CRC specimens, whereas Wip1 exhibits considerable variations among patients, and Wip1 levels are negatively correlated with prognosis. Furthermore, animal experiments showed that the combination of EA and oxaliplatin is more suitable for tumors with high Wip1 expression, suggesting that Wip1 expression may need to be considered when using ATM inhibitors.

*In vivo*, prototype EA is mainly generated from the hydrolysis of ellagitannins derived from foods such as pomegranates, berries, and walnuts. EA is poorly absorbed in the small intestine, EA is poorly absorbed in the small intestine, but LC-MS results have confirmed the presence of EA in the serum of patients treated with ellagitannin-containing Xihuang Pill, indicating the clinical relevance and research significance of exploring the adjuvant anticancer mechanisms of EA. In addition, EA can be metabolized and converted into urolithin A by gut bacteria in the colon. However, humans are classified into three urolithin metabotypes. Individuals with the UM-0 metabotype lack the corresponding gut bacteria and cannot produce urolithin A even after EA intake (Ribeiro et al., 2025). For this subgroup, only EA exerts biological effects following ellagitannin consumption. Given the distinct mechanisms of action between EA and urolithin A, the present study only investigated EA, which constitutes one of the limitations of this work. Future work should further explore the biological activities of EA-derived gut microbial metabolites including urolithin A, to clarify their respective pharmacological roles under different urolithin-metabotype backgrounds.

Although EA has not been used as a single anti-tumor drug in clinical practice, many plant-derived compound formulations containing EA have been widely used to enhance the efficacy of chemotherapy for CRC (Yu and An, 2017; Augustynowicz et al., 2022). This study elucidated the mechanism underlying the combined use of EA and OXA in the treatment of CRC. Considering existing studies on the combination of EA and 5-fluorouracil (Kao et al., 2012), it can be inferred that EA or EA-containing herbal formulas may be suitable for combination with the FOLFOX regimen to improve the prognosis of CRC with Wip1 overexpression. Furthermore, this study was limited to CRC with wild-type *TP53*.

## Data Availability

The datasets generated and/or analyzed during the current study are available in https://figshare.com/s/178239ee51ed3f0f38e5.
