# Supplementary material for: Ellagic acid promoted efficacy of oxaliplatin on wild-type TP53 colorectal cancer via regulating ataxia-telangiectasia mutated protein kinase and Wip1
Source: Front Pharmacol. 2026 Oct 15;17:1944826. doi: 10.3389/fphar.2026.1944826 (PMC13716623; doi:10.3389/fphar.2026.1944826)
Supplement: Supplementary file 1 [file Supplementaryfile1.docx]

**Figure S1**. Results of three independent Annexin V‑PI flow cytometry assays of **Figure 1B**.

**Figure S2**. Results of PI flow cytometric cell cycle analysis, including quantitative cell-cycle distribution data from three independent flow cytometry measurements.

**Figure S3**. The grayscale value analysis of Figure 2D.

**Figure S4**. The grayscale value analysis of Figure 3A.

**Figure S5**. The grayscale value analysis of Figure 3B.

**Figure S6**. Reaction formula for the synthesis of biotin-conjugated EA from EA and biotin.

**Figure S7**. EA directly interacts with ATM in Caco2 and SW48 cells validated by CETSA. Cells with or without EA treatment were heated at a gradient temperature ranging from 37 °C to 68 °C. β-tubulin was used as an internal reference control. All experimental conditions were performed with four independent replicates.

**Figure  S8**. The effect of M6620 on the synergistic effect. (A) M6620 decreased p-ATR levels in ‘EA+OXA’ treated cells. (B) CCK-8 assays of M6620 treated cells. Cells were treated with EA and OXA alone or in combination at the indicated concentrations. Inhibition rates were compared between monotherapy and combination groups. Combination indices were calculated using CalcuSyn software.

**Figure S9**. The grayscale value analysis of Figures 5A-D.

**Table S1**. Summary of patient information included in this study.

| **Characteristic** | **Overall** | **Wip1-low** | **Wip1-high** | **p-value** |
| --- | --- | --- | --- | --- |
| **Sex(%)** |  |  |  | >0.9 |
| man | 43 / 71 (61%) | 21 / 35 (60%) | 22 / 36 (61%) |  |
| woman | 28 / 71 (39%) | 14 / 35 (40%) | 14 / 36 (39%) |  |
| **Location(%)** |  |  |  | <0.001 |
| Left-Sided Colon | 42 / 71 (59%) | 23 / 35 (66%) | 19 / 36 (53%) |  |
| Right-Sided Colon | 29 / 71 (41%) | 12 / 35 (34%) | 17 / 36 (47%) |  |
| **Histology(%)** |  |  |  |  |
| High-grade | 4 / 71 (5.6%) | 0 / 35 (0%) | 4 / 36 (11%) |  |
| Middle-grade | 17 / 71 (24%) | 3 / 35 (8.6%) | 14 / 36 (39%) | 0.3 |
| low-grade | 50 / 71 (70%) | 32 / 35 (91%) | 18 / 36 (50%) |  |
| **Vascular invasion(%)** |  |  |  |  |
| no | 55 / 71 (77%) | 28 / 35 (80%) | 27 / 36 (75%) |  |
| yes | 16 / 71 (23%) | 7 / 35 (20%) | 9 / 36 (25%) | 0.019 |
| **Neurovascular invasion(%)** |  |  |  |  |
| no | 58 / 71 (82%) | 33 / 35 (94%) | 25 / 36 (69%) |  |
| yes | 13 / 71 (18%) | 2 / 35 (5.7%) | 11 / 36 (31%) | 0.6 |
| **MMR(%)** |  |  |  |  |
| dMMR | 17 / 71 (24%) | 9 / 35 (26%) | 8 / 36 (22%) |  |
| pMMR | 54 / 71 (76%) | 26 / 35 (74%) | 28 / 36 (78%) | 0.007 |
| **Age(%)** |  |  |  |  |
| ＜60 | 30 / 71 (42%) | 13 / 35 (37%) | 17 / 36 (47%) |  |
| ≥60 | 41 / 71 (58%) | 22 / 35 (63%) | 19 / 36 (53%) | 0.7 |
| **Level of Expression(%)** |  |  |  |  |
| Wip1-high | 36 / 71 (51%) | 0 / 35 (0%) | 36 / 36 (100%) |  |
| Wip1-low | 35 / 71 (49%) | 35 / 35 (100%) | 0 / 36 (0%) | 0.4 |

**Figure S10**. EA promotes Wip1 ubiquitination while maintaining the HIPK2-Wip1 interaction. Cells were treated with MG132 3h prior to harvest. Co-immunoprecipitation assays were performed in Caco2 and SW48 cell lines. (A) Co-IP results without EA treatment. (B) Co-IP results with EA treatment, showing that the interaction between HIPK2 and Wip1 persists upon EA exposure. (C) Ubiquitination analysis of Wip1, demonstrating that EA enhances the ubiquitination level of Wip1. All experiments were conducted with three independent replicates.

**Figure S11**. LC‑MS analysis of Xihuang Pill and plasma samples from a patient. Peaks below the x-axis represent EA. Notably, EA was undetectable in pretreatment blood samples. (A) Xihuang Pill extract. (B) Plasma sample collected 24 hours after treatment. (C) Plasma sample collected 48 hours after treatment. (D) Pie chart showing the classification of absorbed components from Xihuang Pill.

**Figure S12**. Somatic mutation analysis of representative P2 tumors and the corresponding patient‐derived parental tumors.

**Figure S13**. Detection of Hepatic and Renal Injury in PDX Tumor‑Bearing Mice. (A) Blood was collected from mice before drug administration and on day 10 post‑treatment for measurement of blood urea nitrogen (BUN) and creatinine (Cr) (renal function), together with aspartate aminotransferase (AST) and alanine aminotransferase (ALT) (hepatic function). **P*<0.05 (B) H&E staining was performed to evaluate injuries of renal tubules and hepatic lobules.

**Figure S14**. Statistical analysis of **Figure 8**. Each group was compared with the NC group, and horizontal lines indicate comparisons between other groups.
